# *Mrpl10* and *Tbp* Are Suitable Reference Genes for Peripheral Nerve Crush Injury

**DOI:** 10.3390/ijms18020263

**Published:** 2017-01-27

**Authors:** Yaxian Wang, Qianqian Shan, Yali Meng, Jiacheng Pan, Sheng Yi

**Affiliations:** 1 School of Biology and Basic Medical Sciences, Soochow University, Suzhou 215123, China; wyx1984@ntu.edu.cn; 2 Key Laboratory of Neuroregeneration of Jiangsu and Ministry of Education, Co-Innovation Center of Neuroregeneration, Nantong University, Nantong 226001, China; 18705592415@163.com (Y.M.); kaixinge@126.com (J.P.); 3 Department of Radiotherapy and Oncology, The Affiliated Hospital of Nantong University, Nantong 226001, China; shanqianiqan1986@126.com

**Keywords:** rat peripheral nerve injury, distal nerve stump, dorsal root ganglion, housekeeping gene, geNorm, NormFinder

## Abstract

Peripheral nerve injury triggers the dysregulation of a large number of genes at multiple sites, including neurons, peripheral nerve stump, and the target organ. Housekeeping genes were frequently used as reference genes to normalize the expression values of target genes. Suitable selection of housekeeping genes that are stably expressed after nerve injury minimizes bias elicited by reference genes and thus helps to better and more sensitively reflect gene expression changes. However, many housekeeping genes have been used as reference genes without testing the expression patterns of themselves. In the current study, we calculated the expression stability of nine commonly used housekeeping genes, such as *18S* (18S ribosomal RNA), *Actb* (β-actin), *CypA* (cyclophilin A), *Gapdh* (glyceraldehydes-3-phosphate dehydrogenase), *Hprt* (hypoxanthine guanine phosphoribosyl transferase), *Pgk1* (phosphoglycerate kinase 1), *Tbp* (TATA box binding protein), *Ubc* (ubiquitin C), *YwhaZ* (tyrosine 3-monooxygenase/tryptophan 5-monooxygenase activation), and four newly identified housekeeping genes, including *Ankrd27* (Ankyrin repeat domain 27), *Mrpl10* (mitochondrial ribosomal protein L10), *Rictor* (rapamycin-insensitive companion of mTOR, Complex 2), and *Ubxn 11* (UBX domain protein 11), in both distal sciatic nerve samples and dorsal root ganglion (DRG) samples after sciatic nerve injury. Our results suggested that following peripheral nerve injury, *Mrpl10* and *Tbp* might be used as suitable reference genes for sciatic nerve stump and DRGs, respectively.

## 1. Introduction

Peripheral nerve injury triggers complicated biological changes in many tissues and organs, such as neurons, the injured site, proximal and distal nerve stumps, and the target organ. A series of intricate biological activities happen following peripheral nerve injury, including the disintegration of axon and myelin sheaths, Wallerian degeneration of the distal nerve stump, the migration of macrophages and monocytes to the injured site, the dedifferentiation and proliferation of Schwann cells, and the regrowth and re-myelination of injured axon [1,2,3]. These complex biological processes are accompanied with the dysregulation of numerous genes [4,5].

Emerging studies have been focused on identifying differentially expressed genes, aiming to decipher molecular and cellular changes following peripheral nerve injury. Quantitative real time polymerase chain reaction (qRT-PCR) has been widely used to determine expression patterns of target genes.

qRT-PCR, due to its sensitivity, simplicity, specificity, and high-throughput potential, has rapidly become a popular biological tool for mRNA quantification since its discovery by Kary Mullis in 1985 [6,7]. However, outcomes from qRT-PCR may be significantly affected by inherent sample variations. To minimize experimental errors and to increase the accuracy and reproducibility of qRT-PCR outcomes, the magnitude of absolute measures obtained from qRT-PCR are calibrated and normalized by using an internal control [8]. Housekeeping genes are very often used as reference genes. Housekeeping genes are constitutive genes that are expressed in almost all cell types at relative stable levels [9,10]. Till now, a large number of housekeeping genes has been identified and used [11]. It is worth noting that, in some physiological or pathological conditions, many housekeeping genes may express different expression patterns [12]. Therefore, prior to its potential application, the expression stability of certain housekeeping genes should be validated first. Only housekeeping genes with the lowest variability should be used for qRT-PCR to avoid the bias caused by improper employment of differentially expressed housekeeping genes.

The purpose of the current research was to select suitable reference gene(s) for the study of peripheral nerve crush injury. Nine commonly used housekeeping genes, including *18S* (18S ribosomal RNA), *Actb* (β-actin), *CypA* (cyclophilin A), *Gapdh* (glyceraldehydes-3-phosphate dehydrogenase), *Hprt* (hypoxanthine guanine phosphoribosyl transferase), *Pgk1* (phosphoglycerate kinase 1), *Tbp* (TATA box binding protein), *Ubc* (ubiquitin C), and *YwhaZ* (tyrosine 3-monooxygenase/tryptophan 5-monooxygenase activation); and four recently identified housekeeping genes [13], including *Ankrd27* (Ankyrin repeat domain 27), *Mrpl10* (mitochondrial ribosomal protein L10), *Rictor* (rapamycin-insensitive companion of mTOR, Complex 2), and *Ubxn 11* (UBX domain protein 11), were tested as candidate reference genes. By using qRT-PCR, the expression levels of these housekeeping genes were measured in both distal sciatic nerve stump and dorsal root ganglion (DRG) samples, two main tissues that are critical for the degeneration and regeneration of injured peripheral nerve, at 0, 1, 4, 7, 14, and 21 days post sciatic nerve crush. Obtained Threshold cycle (CT) values were used to evaluate the expression stability of these candidate genes by using geNorm and NormFinder software [14,15].

Compared with sciatic nerve transection, sciatic nerve crush keeps the basal luminal intact and thus injured nerve regrow and regeneration in a relative shorter time period. Therefore, in our current study, we used the distal nerve stump about 3 mm from the crush site, trying to include more biological processes, such as Wallerian degeneration at the distal nerve stump at an early stage post crush and the regeneration and reconnection of growing proximal nerve stump to the distal nerve stump.

Our outcomes suggested that after sciatic nerve crush, *Mrpl10* and *Tbp* were expressed most stably in distal sciatic nerve stump and DRGs, respectively, and therefore could be used as reference genes to study the relative expression levels of target genes following peripheral nerve crush injury.

## 2. Results

### 2.1. Peripheral Nerve Injury Involves Complex Biological Processes

**Figure 1:**
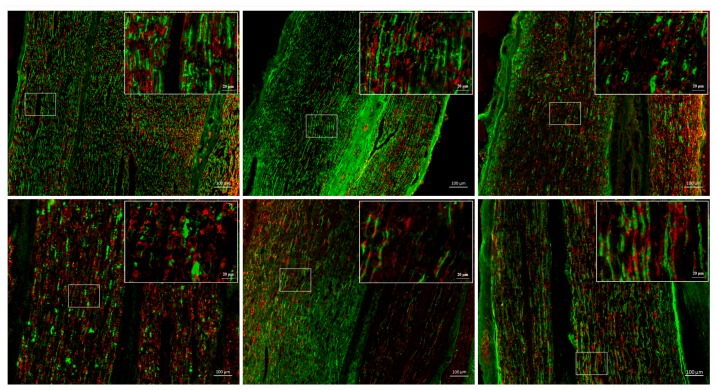
Immunohistochemistry images of distal sciatic nerve at 0, 1, 4, 7, 14, and 21 days post nerve crush. Distal sciatic nerves were stained with anti-NF200 (**green**) and anti-S100 (**red**). Scale bar = 100 μm. Images in the box to the upper right corner represented the higher magnifications of the boxed areas. Scale bar = 20 μm.

Genetic changes and morphological changes are normally highly related with each other. Therefore, in the current study, immunochemical staining of rat distal nerve stump was firstly performed to observe injury induced morphological changes (Figure 1). Rat distal nerve stump was co-immunostained with antibodies against NF-200, a neuronal marker, and S100, a Schwann cell marker. Immunostaining with NF-200 (in green) and S100 (in red) showed that Schwann cells wrapped around axon tightly in the normal sciatic nerve stump. At one day post nerve crush, in the distal nerve stump, the alignment of NF-100 and S100 could still be observed but seemed less organized. At four days post crush, NF-100 and S100 seemed fragmented and scrambled, suggesting that there existed lots of axon and Schwann cell debris and the distal nerve stump underwent degeneration. The fragmentation of axons and Schwann cells became more severe at seven days post crush. At longer time periods, the orientation and arrangement of NF-200 and S100 signals started to recover back to normal conditions, indicating that injured axon began to regrow and reconnect with the distal stump and Schwann cells started to spiral around axon to form myelin sheaths. Outcomes from immunochemical staining suggested that, in the distal sciatic nerve stump, the degeneration process occurs violently and reaches a peak at seven days post crush and then nerve repair and regeneration process starts from 14 days post nerve crush.

The observed dramatic morphological changes suggested that biological events following peripheral nerve injury are very complicated and there may exist significant alterations in the genetic level. Therefore, it is possible that following peripheral nerve injury, the expression levels of many generally used housekeeping genes may be altered. Consequently, gene expression stabilities of some commonly used housekeeping genes were detected.

### 2.2. Mrpl10 Is the Most Stable Expressed Reference Gene in Distal Sciatic Nerve Following Peripheral Nerve Injury

To identify the expression stabilities of housekeeping genes, firstly, the CT values of these 13 selected housekeeping genes in distal sciatic nerve were determined by qRT-PCR (Table 1). Among these 13 genes, *18S*, as a ribosomal RNA, undoubtedly had the highest expression levels (the lowest CT values). *Actb*, the gene coding for cell cytoskeletal protein β-actin had the second largest expression levels. The CT values of other housekeeping genes were among 20–36.

**Figure 2:**
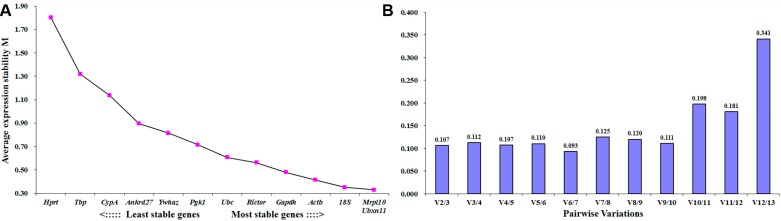
geNorm determined expression stability of candidate housekeeping genes in distal sciatic nerve samples. (**A**) Average expression stability (M) values of tested housekeeping genes. The lower M value, the higher the gene stability; (**B**) The determination of the optimal number of control genes for accurate normalization by using pairwise variation (V) analysis. Histograms indicated the variation values when other endogenous housekeeping genes were added according to the stability order in (**A**).

Raw expression data of each housekeeping gene were then analyzed by geNorm software. Gene stability was inversely proportional to geNorm calculated M value. Our results suggested that besides *Hprt*, every other tested housekeeping genes had an M value less than threshold 1.5, and could be considered as relatively constant [14]. Among all tested housekeeping genes, *Mrpl10* and *Ubxn 11* were the two most stable ones at different time points post injury (Figure 2A). Besides M value, geNorm software was also used to calculate pairwise variations (V_n/n+1_), an index that determines the optimal number of control genes for normalization. The variation value of V_2/3_ was 0.107, far below the cutoff value 0.15, suggesting that combination of *Mrpl10* and *Ubxn 11* was sufficient for normalization (Figure 2B).

NormFinder software was also applied to determine gene expression stability. By using a model-based approach, we obtained a ranking order of all tested housekeeping genes (Table 2). Similar as outcomes from geNorm analysis, *Mrpl10* was the most stable gene. However, *Ubxn 11* only ranked ninth among these 13 tested housekeeping genes according to this program.

### 2.3. Tbp Is the Most Stable Expressed Reference Gene in DRGs Following Peripheral Nerve Injury

Besides the nerve stump, following peripheral nerve injury, DRGs is also commonly affected and thus has been widely studied. Considering the importance of DRGs in nerve injury and regeneration, we further tested the expression values of these housekeeping genes in DRG samples (Table 3). Similarly, *18S* had the lowest CT values and the CT values of *18S* in DRG samples seemed to be ever lower than those in distal sciatic nerve stump samples. Additionally, the CT values of most other housekeeping genes (except for *Ubxn11*) in DRGs were relatively lower than those in distal nerve stump. The highest CT value for these housekeeping genes was ~30.

**Figure 3:**
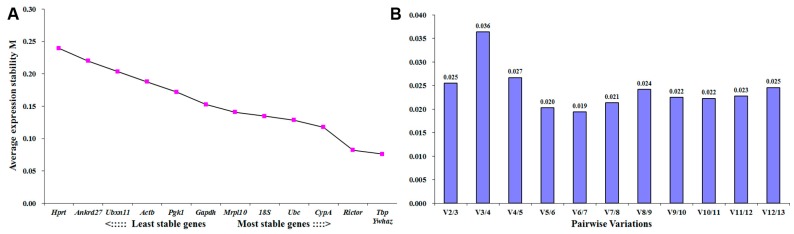
geNorm determined expression stability of candidate housekeeping genes in dorsal root ganglion (DRG) samples. (**A**) Average expression stability (M) values of tested housekeeping genes. The lower M value, the higher the gene stability; (**B**) The determination of the optimal number of control genes for accurate normalization by using pairwise variation (V) analysis. Histograms indicated the variation values when other endogenous housekeeping genes were added according to the stability order in (**A**).

Identical to genes in distal nerve stump, raw expression data were analyzed by geNorm and NormFinder software. Outcomes from geNorm analysis suggested that *Tbp* and *Ywhaz* had the lowest M value (Figure 3A) and the pairwise variation of *Tbp* and *Ywhaz* was only 0.025 (Figure 3B), indicating that these two housekeeping genes were suitable candidates for reference genes in DRG samples. Outcomes from NormFinder analysis showed that *Tbp* ranked first while *Ywhaz* ranked fourth among these candidate housekeeping genes (Table 4).

## 3. Discussion

Recently, qRT-PCR has been performed more and more widely in biological studies. Additionally, as the pervasive applications of high-throughput technologies (e.g., microarray, RNA sequencing, and DNA sequencing), qRT-PCR analysis is generally used as the golden standard to validate the accuracy of outcomes from high-throughput technologies. Therefore, the accuracy of qRT-PCR analysis itself becomes of critical importance. Reliable and reproducible quantifications of target genes require well designed assays and protocols, high quality RNA samples, high efficiency amplification enzymes, suitable primer pairs, and appropriate normalization methods. Due to its convenience and easy accessibility, the housekeeping gene has been frequently applied as an internal control to diminish deviation and to provide more accurate outcomes [16,17].

With the development qRT-PCR analysis and especially the wide spread applications of high-throughput technologies, many genes that were essential for cellular functions and relatively equally expressed in all cell types were identified as housekeeping genes and then used as reference genes. It is noteworthy that the expression patterns of many of these identified housekeeping genes might be altered under lots of specific treatments or physiological/pathological conditions. For instance, when studying RNA sequencing data obtained from crushed sciatic nerve stump, we found that canonical signaling pathways “actin cytoskeleton signaling” and “regulation of actin-based motility by Rho” were highly activated. The expression levels of *Actb*, a widely used housekeeping gene, were upregulated more than two-fold at one and four days post injury (unpublished data). There exists no single gene that expresses at a constant level and can be used as a universal reference gene [18,19].

In the peripheral nerve system, qRT-PCR has been routinely used to identify the expression levels of target genes in the nerve stump and DRGs at different time points post injury to investigate sciatic nerve injury mediated genetic changes. Our morphological outcomes (Figure 1) and previous observations from microarray and RNA sequencing suggested that dramatic changes and significantly dysregulated genes exist in both sciatic nerve stump and DRGs [20,21,22,23,24,25]. The expression patterns of numerous housekeeping genes in the nerve stump (Table S1) and DRG samples (Table S2) further suggested that even housekeeping genes were differentially expressed post injury. A suitable selection of stable expressed housekeeping gene(s), therefore, is of great importance. In an earlier study, Gambarotta et al. measured the CT values of many housekeeping genes in the distal nerve stump using both sciatic nerve crush model and sciatic nerve transection model and concluded that *Ankrd27* and *Rictor* could be used as reference genes for qRT-PCR normalization [13]. Previous high-sequencing outcomes suggested that the biological events happened post nerve crush and transection might not be totally identical [20,21]. Therefore, in the current study, to further identify stable housekeeping genes post crush, we studied the expression stability of housekeeping genes only in crushed distal sciatic nerve at different time points. Our outcomes, however, showed that *Mrpl10*, instead of *Ankrd27* and *Rictor*, was the most stable expressed housekeeping genes at 0, 1, 4, 7, 14, and 21 days post injury. In another study, Bangaru et al. performed spinal nerve ligation, detected the relative stability of *Mapk6*, *Gapdh*, *Tubb5*, *Hprt1*, *18S*, *Actb*, and *Tubb3*, and then showed that *Mapk6* and *Gapdh* were the most stable housekeeping genes [26]. In our current study, we also measured the expression stability of *Gapdh*, *Hprt1*, *18S*, and *Actb*. However, our results suggested that *18S* was more stable than *Gapdh* in the sciatic nerve stump and DRGs following nerve crush. These inconsistent observations increased the complexity of choosing reference genes and implied that one might need to select stably expressed housekeeping genes according to experimental designs prior to performing qRT-PCR.

## 4. Materials and Methods

### 4.1. Animal Surgery

A total of 36 adult female Sprague-Dawley (SD) rats (180–220 g) were randomly separated into six groups and underwent surgery of sciatic nerve crush as previously described [21]. Briefly, after anaesthetization, rat left sciatic nerves at 10 mm above the bifurcation into the tibial and common fibular nerves were crushed. At 1, 4, 7, 14, and 21 days post nerve injury, animals were killed by decapitation. The lumbar 4–6 (L4–6) DRGs and the sciatic nerve segments of 5 mm in length in the distal stump were harvested from rats in different groups. The rats in the 0-day group underwent sham-surgery. Animals were purchased from the Experimental Animal Center of Nantong University (animal license No. SCXK [Su] 2014-0001 and SYXK [Su] 2012-0031). Procedures of animal surgery were performed according to Institutional Animal Care guideline of Nantong University and ethically approved by the Administration Committee of Experimental Animals, Jiangsu Province, China.

### 4.2. Immunochemical Staining

Collected distal sciatic nerve was intracardially perfused with 4% freshly depolymerized, neutral-buffered paraformaldehyde. After fixation, tissue samples were embedded in Tissue-Tek Optimal Cutting Temperature (O.C.T) compound and sectioned on a cryostat microtome (Leica, Germany) at 12 μm thick. Tissue slices were mounted on microscope slides, and subjected to immune-histochemistry primary antibodies, mouse anti NF-200 (1:200, N2912, Sigma, St. Louis, MO, USA) and rabbit anti-S100 (1:200, Sigma S2644) overnight at 4 °C followed by incubation with secondary antibodies, goat-anti-mouse IgG labeled with Alexa-flour 488 (1:400, Abcam, Cambridge, MA, USA), and goat-anti-rabbit IgG labeled with Alexa-flour 555 (1:500, Abcam), for 2 h at room temperature. Images were taken under fluorescence microscopy (ZEISS, Jena, Germany).

### 4.3. RNA Extraction

RNA samples were extracted from distal sciatic nerve and DRGs using Trizol Reagent (Life Technologies, Carlsbed, CA, USA) according to manufacturer’s instructions and then purified with RNeasy spin columns (Qiagen, Valencia, CA, USA) to remove remaining DNA. The quantity of isolated RNA samples was evaluated with a NanoDrop ND-1000 spectrophotometer (Infinigen Biotechnology Inc., City of Industry, CA, USA).

### 4.4. qRT-PCR

1 μg RNA samples were reverse transcribed to cDNA with the Prime-Script reagent Kit (TaKaRa, Dalian, China) and qRT-PCR was performed with SYBR Green Premix Ex Taq (Roche, Basel, Switzerland) on an Applied Biosystems StepOne real-time PCR System according to standard protocols. The thermocycler program was 10 min at 95 °C; 40 cycles of 10 s at 95 °C, 30 s at 60 °C. The quality of qRT-PCR outcomes was confirmed by the observation of a single peak melt curve, which represented a single product. Primer pairs were synthesized by Invitrogen Life Technologies either according to previous publications [13,27,28,29] or new designedly by Primer Express^®^ software (Applied Biosystems, Foster City, CA, USA). The amplification of primer pairs was determined by serial dilutions and primers with amplification efficiencies between 90% and 110% were chosen (Table 5, Figure S1).

### 4.5. Bioinformatic Analysis

For each housekeeping gene, the CT values obtained in each sample from qRT-PCR were used to calculate comparative 2^−∆*C*t^, in which ∆*C*_t_ equaled to highest *C*_t_ value minus sample *C*_t_ value. The sample with the lowest *C*_t_ value (corresponding to the highest expression level) had a ∆*C*_t_ of 0 while other samples had ∆*C*_t_ greater than 0. Calculated values of 2^−∆*C*t^ were applied to geNorm [14] and NormFinder analysis [15] by using Microsoft Excel add-in software. geNorm software calculated an average expression stability (M) value which was inversely proportional to the expression stability of tested gene. Moreover, geNorm software provided a normalization factor which was derived from the geometric mean of the included genes and gave the minimum number of control genes for normalization. A normalization factor less than 0.15 indicated that the number of reference genes met requirements and no housekeeping gene needed to be added [26]. NormFinder software calculated the intra-group and inter-group variations and provided the expression stability value of each tested gene [26].

## 5. Conclusions

In conclusion, by the joint use of geNorm and NormFinder software, we calculated the expression variations of 13 commonly used housekeeping genes. Analysis from both geNorm and NormFinder software suggested that housekeeping genes, *Mrpl10* and *Tbp*, had the highest expression stabilities in distal sciatic nerve stump and DRGs, respectively. Our current study might contribute to the identification of reference genes for peripheral nerve crush injury. More importantly, we hope our study could signal the importance of selecting suitable internal controls for qRT-PCR qualification.

## Figures and Tables

**Table 1:** Raw CT values of distal sciatic nerve samples at 0, 1, 4, 7, 14, and 21 days post sciatic nerve crush. (CT values were presented as means ± SEM).

Gene Symbol	0 Day	1 Day	4 Days	7 Days	14 Days	21 Days
*18S*	9.27 ± 0.77	8.18 ± 0.28	5.33 ± 0.26	5.43 ± 0.34	5.51 ± 0.30	8.67 ± 0.29
*Actb*	19.82 ± 0.28	17.90 ± 0.09	16.02 ± 0.10	16.44 ± 0.16	16.89 ± 0.08	19.37 ± 0.39
*Ankrd27*	28.97 ± 0.74	29.13 ± 0.36	24.36 ± 0.19	24.37 ± 0.18	24.15 ± 0.18	28.30 ± 0.59
*CypA*	25.46 ± 0.70	24.21 ± 0.54	17.87 ± 0.32	18.26 ± 0.33	19.14 ± 0.55	26.19 ± 0.70
*Gapdh*	21.05 ± 0.46	19.95 ± 0.02	17.73 ± 0.12	18.77 ± 0.08	19.04 ± 0.12	21.68 ± 0.08
*Hprt*	35.81 ± 0.22	30.93 ± 0.04	21.74 ± 0.74	21.77 ± 0.85	22.01 ± 0.69	33.10 ± 0.84
*Mrpl10*	28.95 ± 0.64	27.90 ± 0.09	25.41 ± 0.24	25.86 ± 0.22	26.05 ± 0.12	28.81 ± 0.12
*Pgk1*	25.62 ± 0.59	23.26 ± 0.45	20.19 ± 0.47	21.18 ± 0.51	22.12 ± 0.49	25.61 ± 0.47
*Rictor*	26.93 ± 0.00	26.64 ± 0.29	24.92 ± 0.16	24.87 ± 0.19	24.84 ± 0.17	26.53 ± 0.22
*Tbp*	32.71 ± 0.12	30.54 ± 0.54	24.77 ± 0.43	24.75 ± 0.44	24.69 ± 0.44	32.57 ± 0.61
*Ubc*	22.94 ± 0.00	22.84 ± 0.21	20.79 ± 0.22	21.13 ± 0.21	21.29 ± 0.28	22.43 ± 0.24
*Ubxn11*	31.07 ± 0.56	30.54 ± 0.20	28.21 ± 0.22	28.03 ± 0.16	28.03 ± 0.12	30.94 ± 0.01
*Ywhaz*	24.61 ± 0.85	24.06 ± 0.21	20.21 ± 0.13	20.07 ± 0.01	20.29 ± 0.13	25.34 ± 0.45

**Table 2:** NormFinder determined stability values of candidate housekeeping genes in distal sciatic nerve samples. Stability values of tested housekeeping genes were calculated by NormFinder software and then ranked. The lower the stability value, the higher the gene stability.

Ranking Order	Gene Name	Stability Value
1	*Mrpl10*	0.053
2	*18S*	0.058
3	*Rictor*	0.097
4	*Ubc*	0.105
5	*Actb*	0.111
6	*Ywhaz*	0.112
7	*Hprt*	0.120
8	*CypA*	0.121
9	*Ubxn11*	0.125
10	*Ankrd27*	0.145
11	*Tbp*	0.160
12	*Gapdh*	0.202
13	*Pgk1*	0.214

**Table 3:** Raw CT values of dorsal root ganglion (DRG) samples at 0, 1, 4, 7, 14, and 21 days post sciatic nerve crush. (CT values were presented as means ± SEM).

Gene Name	0 Day	1 Day	4 Days	7 Days	14 Days	21 Days
*18S*	6.17 ± 0.23	5.72 ± 0.15	5.75 ± 0.14	5.78 ± 0.17	5.88 ± 0.09	5.90 ± 0.17
*Actb*	18.52 ± 0.03	17.94 ± 0.03	17.87 ± 0.10	17.70 ± 0.06	17.83 ± 0.05	17.87 ± 0.06
*Ankrd27*	24.20 ± 0.15	24.56 ± 0.04	24.06 ± 0.17	23.84 ± 0.15	23.87 ± 0.11	23.93 ± 0.03
*CypA*	17.81 ± 0.17	17.57 ± 0.06	17.67 ± 0.12	17.84 ± 0.13	17.89 ± 0.10	17.91 ± 0.05
*Gapdh*	17.38 ± 0.76	17.34 ± 0.67	17.16 ± 0.56	17.13 ± 0.60	17.22 ± 0.61	16.99 ± 0.76
*Hprt*	23.42 ± 0.12	22.96 ± 0.01	22.48 ± 0.16	22.27 ± 0.08	22.42 ± 0.08	22.77 ± 0.13
*Mrpl10*	25.90 ± 0.04	25.54 ± 0.07	25.49 ± 0.08	25.63 ± 0.17	25.95 ± 0.02	25.90 ± 0.06
*Pgk1*	21.10 ± 0.07	20.86 ± 0.10	20.64 ± 0.05	20.35 ± 0.12	20.77 ± 0.09	20.93 ± 0.00
*Rictor*	25.25 ± 0.54	25.32 ± 0.57	25.14 ± 0.62	25.23 ± 0.62	25.17 ± 0.55	25.29 ± 0.49
*Tbp*	26.06 ± 0.13	25.90 ± 0.06	25.89 ± 0.04	25.83 ± 0.20	25.85 ± 0.14	25.92 ± 0.02
*Ubc*	21.86 ± 0.12	21.52 ± 0.08	21.61 ± 0.13	21.73 ± 0.07	21.91 ± 0.03	21.91 ± 0.03
*Ubxn11*	30.23 ± 0.33	30.21 ± 0.29	29.60 ± 0.39	29.65 ± 0.21	29.74 ± 0.16	30.18 ± 0.43
*Ywhaz*	18.94 ± 0.02	18.92 ± 0.09	18.90 ± 0.06	18.91 ± 0.06	18.94 ± 0.03	18.88 ± 0.03

**Table 4:** NormFinder determined stability values of candidate housekeeping genes in DRG samples. Stability values of tested housekeeping genes were calculated by NormFinder software and then ranked. The lower the stability value, the higher the gene stability.

Ranking Order	Gene Name	Stability Value
1	*Tbp*	0.026
2	*18S*	0.058
3	*Rictor*	0.066
4	*Ywhaz*	0.070
5	*Pgk1*	0.083
6	*Gapdh*	0.083
7	*Actb*	0.089
8	*Mrpl10*	0.103
9	*Ubc*	0.107
10	*CypA*	0.114
11	*Ubxn11*	0.116
12	*Ankrd27*	0.140
13	*Hprt*	0.144

**Table 5:** Primer sequences of candidate housekeeping genes evaluated in this study.

Symbol	Accession Number	Primer Sequences	Amplicon Size	Efficiency	Reference
*18S*	X01117.1	Sense: ACTCAACACGGGAAACCTCA Antisense: AATCGCTCCACCAACTAAGA	114	96%	[26]
*Actb*	NM_031144.2	Sense: AGGCCAACCGTGAAAAGATG Antisense: ACCAGAGGCATACAGGGACAA	101	94%	[27]
*Ankrd27*	NM_001271264.1	Sense: CCAGGAGACAGAACACGAGG Antisense: CCCCTGGGTTAATGAGGCAA	119	91%	*
*CypA*	NM_017101.1	Sense: CCAAACACAAATGGTTCCCAGT Antisense: ATTCCTGGACCCAAAACGCT	135	101%	[26]
*Gapdh*	NM_017008.4	Sense: CAACTCCCTCAAGATTGTCAGCAA Antisense: GGCATGGACTGTGGTCATGA	118	92%	[28]
*Hprt*	NM_012583.2	Sense: TCCCAGCGTCGTGATTAGTGA Antisense: CCTTCATGACATCTCGAGCAAG	152	91%	[26]
*Mrpl10*	NM_001109620.1	Sense: CTCCTCCCAAGCCCCCCAAG Antisense: CAGACAGCTATCATTCGGTTGTCCC	97	92%	[13]
*Pgk1*	NM_053291.3	Sense: ATGCAAAGACTGGCCAAGCTAC Antisense: AGCCACAGCCTCAGCATATTTC	104	91%	[28]
*Rictor*	XM_001055633.3	Sense: GAGGTGGAGAGGACACAAGCCC Antisense: GGCCACAGAACTCGGAAACAAGG	81	91%	[13]
*Tbp*	NM_001004198.1	Sense: TGGGATTGTACCACAGCTCCA Antisense: CTCATGATGACTGCAGCAAACC	131	95%	[28]
*Ubc*	NM_017314.1	Sense: ATCTAGAAAGAGCCCTTCTTGTGC Antisense: ACACCTCCCCATCAAACCC	51	90%	[26]
*Ubxn11*	NM_138853.2	Sense: GCGAGACTGGATGAAGGCCAAG Antisense: CCCTCCACCACCAGCTCACTC	120	110%	[13]
*Ywhaz*	NM_013011.3	Sense: GATGAAGCCATTGCTGAACTTG Antisense: GTCTCCTTGGGTATCCGATGTC	117	94%	[28]

* Designed by Primer Express^®^ software (v3.0.1, ThermoFisher Scientific, Waltham, MA, USA).

## Supplementary Materials

Supplementary materials can be found at www.mdpi.com/1422-0067/18/2/263/s1.
