# Supplementary material for: Mrpl10 and Tbp Are Suitable Reference Genes for Peripheral Nerve Crush Injury
Source: Int J Mol Sci. 2017 Jan 27;18(2):263. doi: 10.3390/ijms18020263 (PMC5343799; doi:10.3390/ijms18020263)
Supplement: Supplementary file 1 [file ijms-18-00263-s001.pdf]

Supplementary Materials: *Mrpl10* and *Tbp* Are Suitable Reference Genes for Peripheral Nerve Crush Injury

Yaxian Wang, Qianqian Shan, YaliMeng, Jiacheng Pan and Sheng Yi

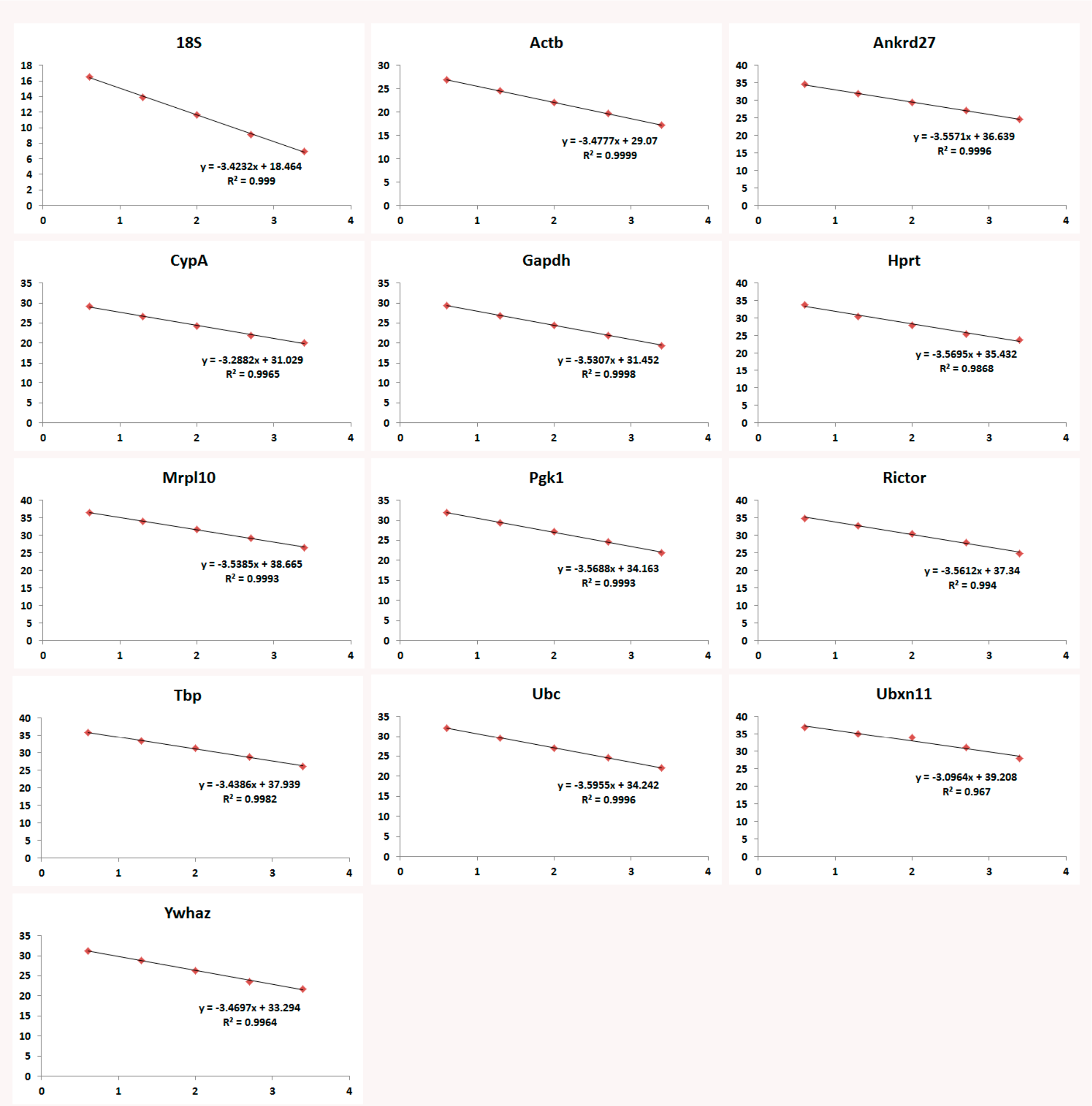

Figure S1. Standard curves for the efficiency of primer pairs. Distal sciatic nerve stumps at 0, 1, 4, 7, 14, and 21 days post injury were mixed together and then diluted at a 5-fold serial dilutions to determine PCR efficiency.

Table S1. Expression patterns of housekeeping genes in crushed nerve stump from previous obtained deep sequencing.(The expression levels of housekeeping genes at different time points post injury were compared with control (0 h) and fold changes were listed in the table.)

Gene Symbol	1 Day	4 Days	7 Days	14 Days
Actb	2.10	2.16	1.51	1.46
Ankrd27	1.79	1.57	1.43	1.41
Cypa	1.89	1.83	1.18	−0.95
Gapdh	1.72	1.05	−0.58	−0.60
Hprt1	3.02	2.96	1.90	1.93
Mrpl10	1.46	1.30	1.03	1.11
Pgk1	3.80	2.60	1.34	1.17
Rictor	−0.93	1.03	−0.99	1.01
Tbp	2.02	1.59	1.53	1.28
Ubc	1.45	1.35	1.27	1.49
Ubxn11	1.65	2.02	1.88	1.28
Ywhaz	1.88	1.91	1.54	1.53

Table S2. Expression patterns of housekeeping genes in DRGs after nerve crush injury from previous obtained deep sequencing.(The expression levels of housekeeping genes at different time points post injury were compared with control (0 h) and fold changes were listed in the table.)

Symbol	3 h	9 h	1 Day	4 Days	7 Days
Actb	1.01	1.05	1.08	1.04	1.04
Ankrd27	−0.95	−0.92	1.06	−0.96	1.05
Cypa	−0.94	1.04	−0.91	−0.95	−0.97
Gapdh	−0.95	−0.98	−0.90	−0.90	−0.96
Hprt1	−0.94	1.11	1.08	1.15	1.12
Mrpl10	−0.95	−0.96	−0.87	−0.87	−0.92
Pgk1	−0.98	1.03	1.10	1.15	1.24
Rictor	1.15	1.13	1.14	1.13	−0.99
Tbp	1.05	1.08	1.13	1.13	1.03
Ubc	1.05	1.10	−0.92	−0.95	1.01
Ubxn11	1.31	1.10	1.08	1.27	1.37
Ywhaz	1.03	1.00	1.06	1.05	1.02
